# Improved quality of recommendations after sentinel event analysis with recommendation improvement matrix training: a before-and-after study at an international patient safety conference

**DOI:** 10.1136/bmjopen-2025-101743

**Published:** 2025-11-12

**Authors:** Peter de Feiter, Annelies Visser, Alia Al Baharnah, Rabab Alkutbe, Tinka Bakker, Ali Asery, Dave Dongelmans

**Affiliations:** 1 Intensive Care, Amsterdam UMC Locatie AMC, Amsterdam, North Holland, Netherlands; 2 Surgery, Amsterdam UMC Locatie AMC, Amsterdam, Netherlands; 3 Saudi Patient Safety Center, Riyadh, Saudi Arabia; 4 Amsterdam UMC Locatie AMC, Amsterdam, Netherlands

**Keywords:** Quality in health care, Quality Improvement, Safety, Adverse events

## Abstract

**Abstract:**

**Objective:**

To evaluate the effectiveness of the recommendation improvement matrix (RIM) methodology for improving the quality of recommendations resulting from sentinel event analysis, where we hypothesise that the use of the RIM methodology leads to better quality recommendations.

**Design:**

A before-and-after analysis of the quality of the formulated recommendations after sentinel event analysis.

**Setting:**

The study was carried out during the 2023 Saudi Patient Safety Centre International Patient Safety Conference.

**Participants:**

36 conference participants, including nurses, medical doctors, pharmacists, dentists, general practitioners and quality officers.

**Interventions:**

RIM methodology training during a structured 3-hour workshop.

**Main outcome measures:**

The primary outcome was the proportion of recommendations that using the χ^2^ test, passed the RIM filter criteria before and after training. Secondary outcomes included changes in recommendation categorisation within the matrix and participant ratings of feasibility and usability on a five-point Likert scale using a t-test for comparison.

**Results:**

Prior to training, 49 recommendations were generated, of which 63.3% met the filter criteria. Post-training, the proportion of recommendations passing the filter increased significantly to 83% (p=0.00543). Adjustments to recommendations primarily improved alignment with the filter criteria, though limited improvements were observed in matrix categorisation. Participants rated the methodology’s feasibility and usability highly, with average scores of 4.39/5 and 4.43/5, respectively. However, 46% expressed a need for additional training, particularly on the matrix application.

**Conclusions:**

The RIM methodology significantly improves the quality of recommendations following sentinel event analyses. To enhance its impact, further training focusing on matrix application is necessary. Incorporating the methodology into healthcare education and professional development could strengthen patient safety practices.

**STRENGTHS AND LIMITATIONS OF THIS STUDY**

- The study employed a standardised case, enabling robust before-and-after comparisons. Participants’ feedback provided valuable insights into the methodology’s practicality.

- The small sample size and diverse professional backgrounds may have influenced the results. Further research is needed to evaluate the methodology’s effectiveness in broader healthcare settings.

## Introduction

 Sentinel events, or adverse events, are defined as unintended patient safety events that result in death, permanent harm or severe temporary harm.^1 2^ They are of great concern in healthcare because the consequences in terms of suffering for patients and their loved ones are immense, the costs are high, and the same sentinel events keep recurring.^3^,^5^ Healthcare providers are second victims of such events, which can lead to various effects on the mental healthcare of personnel. For these reasons, it is standard practice to analyse sentinel events and introduce recommendations to prevent new adverse events. In recent decades, there has been an increasing focus on the development of sentinel event analyses and the effects of the resulting recommendations.^46^,^10^ Many studies conclude that recommendations based on sentinel event analyses have only a limited impact on improving quality and safety in health care.^6 9 10^ A lack of professionalisation of analyses and a lack of standardisation may play a role in this, but the quality of recommendations also sometimes leaves much to be desired.^4 6 8^ Others point to problems with the implementation of recommendations and make suggestions for this, in which continuity in management and a broader focus on causal factors may be important.^7^ Despite this, the same sentinel events continue to occur. In previous research, our group analysed sentinel events and the recommendations for improvement in all Dutch university hospitals. We concluded that there was a large variety in formulating and selecting recommendations after sentinel events. Standards for quality assessment and selection of recommendations following the analyses of sentinel events were lacking.^11 12^

**Figure 1:**
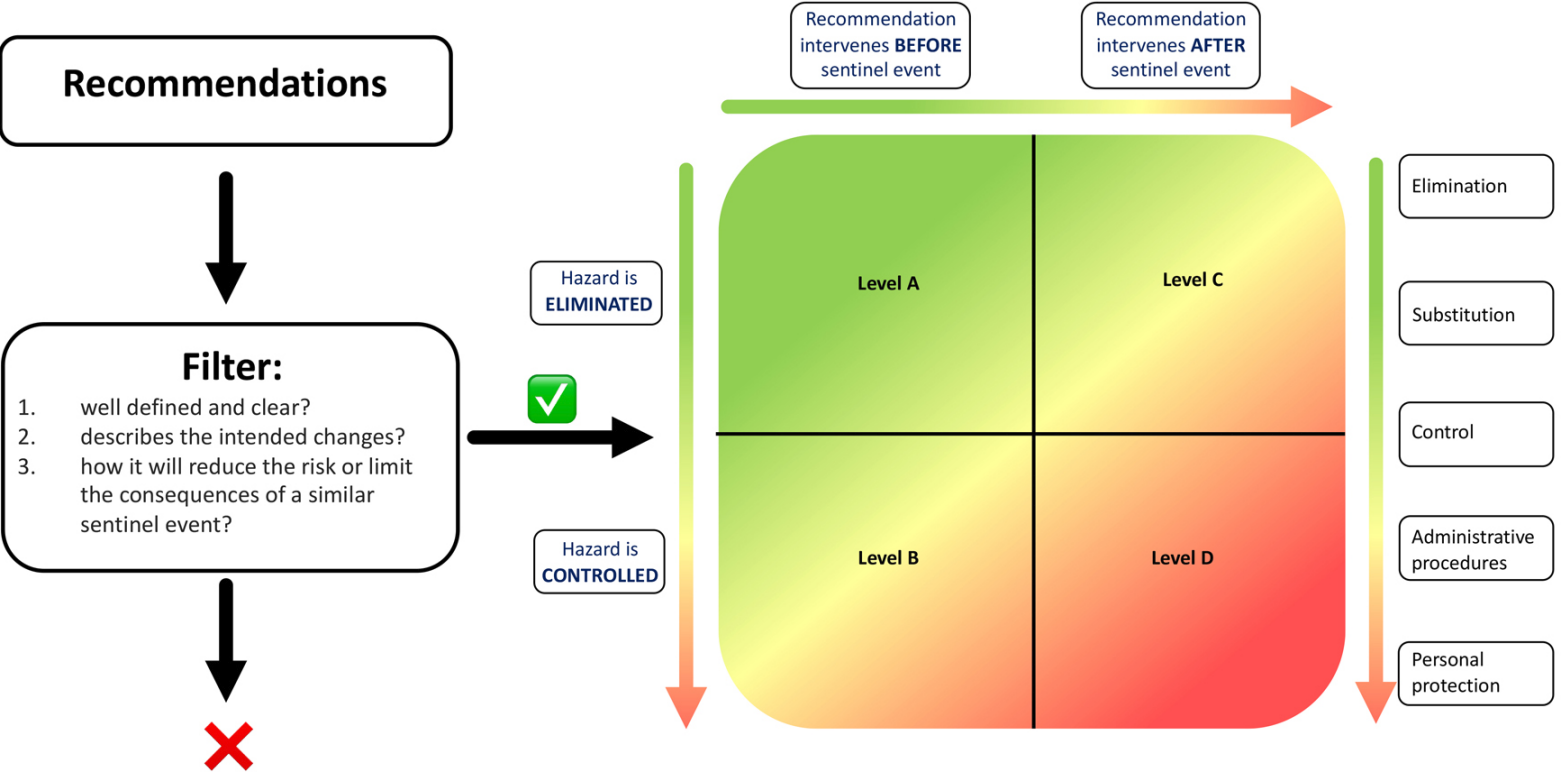
RIM methodology: all recommendations are assessed primarily against the three filter criteria. Only recommendations that meet these criteria are subsequently evaluated using the RIM along the two axes. RIM, recommendation improvement matrix.

There are several methods available to prioritise and select recommendations based on the assessment of their quality following analysis of adverse events. These methods were primarily developed outside healthcare.^13 14^ None of these methods is user-friendly or validated to prioritise recommendations based on objective features.^15^ To assess the quality of recommendations from Dutch perioperative sentinel event analyses, we developed a new method that evaluates recommendations meeting the above-mentioned criteria. In previous publications, we have described how we developed the methodology and which methods developed outside of healthcare served as a basis for it.^15^,^18^ Our method was used to assess the quality of recommendations from Dutch perioperative sentinel event analysis reports.^16 19^ The so-called recommendation improvement matrix (RIM) methodology consists of three filter criteria and the RIM (figure 1). The filter consists of three questions, which must be answered positively: (1) Is the recommendation well-defined and clear? (2) Does the recommendation outline the intended improvement? (3) Does the recommendation specify how it will reduce or mitigate the consequences of a similar sentinel event in the future?

Only recommendations that meet these criteria are subsequently evaluated using the RIM along two axes. The first axis (X-axis) is: does the recommendation prevent the sentinel event from occurring again, or does the recommendation focus on mitigating the effects of the sentinel event? The second axis (Y-axis) is the likelihood of the recommendation being able to achieve the effect which is defined. The likelihood that an administrative recommendation is effective is lower than the elimination of the true reason for the occurrence of a sentinel event.

We evaluated the applicability of this methodology by analysing 115 Dutch reports on perioperative sentinel events in which a total of 161 recommendations were made. The RIM proved useful for grading recommendations and was helpful in both designing and selecting recommendations.^16^ It was also concluded that the use of the RIM methodology resulted in a fair interobserver agreement (interclass correlation coefficient 0.38; 95% CI 0.20 to 0.63) and the inspectors involved in the study (n=4) of the Dutch Health and Youth Care Inspectorate judged that the RIM is user-friendly, understandable and does not take much time. More importantly, they found it potentially useful not only in their own practice but also as a tool for analysing teams to grade their own recommendations.^16^ Although the RIM methodology, according to this study, is promising, the effect of its use on the quality of improvement measures has not yet been further studied in a structured way, nor has the impact of structured training in the RIM methodology itself on the ability of healthcare professionals to formulate higher-quality recommendations been extensively evaluated, and it was assumed that the tool could be mastered quickly, but whether targeted training, including an individual objective assessment regarding mastery of the method, is necessary to ensure a consistent and correct application has not been investigated before and certainly not in a multidisciplinary context.

The aim of the study was therefore to analyse whether, in a fictitious setting involving a presented sentinel event, participants in a workshop, in which they were trained in the RIM methodology, were able to generate higher quality recommendations using this methodology compared with not using it, as well as to analyse the effects of the training itself, including examination of participants' perceptions of the feasibility and usefulness of the RIM methodology in a healthcare setting.

By addressing these objectives, this study contributes to improving the design of optimal recommendations by integrating the RIM methodology into the routine analysis of sentinel events, which also contributes to safety in healthcare.

## Method

A before-and-after analysis of the quality of the formulated recommendations after sentinel event analysis was conducted during the 2023 Saudi Patient Safety Centre International Patient Safety Conference.

The RIM methodology was trained during a structured 3-hour workshop. For the workshop, the authors used a real sentinel event, as described in online supplemental appendix S1. Conference participants were invited to attend the workshop, and no selection criteria were applied in the recruitment process. The participants were nurses, medical doctors, pharmacists, dentists, general practitioners and quality officers. All participants in the workshop were asked about their professional background.

The primary goal was to assess the effect of the RIM methodology in a before-and-after training setting, with the aim of analysing its impact on the formulation of higher-quality recommendations, where we hypothesise that the use of the RIM methodology leads to better quality recommendations. The before-and-after training analysis, in which the recommendations formulated by the subgroups before and after training are qualitatively assessed by an expert panel consisting of two experienced trainers in the RIM methodology. The panel uses the RIM methodology to analyse the differences in the quality of recommendations before and after the training. The quality of the recommendations is expressed in the (1) filter criteria and (2) level and category according to the RIM.

The secondary goal was an analysis of the effect of the training itself through an assessment in which the responses of individual participants are compared with the answers of the expert panel. We hypothesised that the RIM methodology was easy to learn and that the training was usable and feasible.

For the workshop, the authors used a real sentinel event, as described in online supplemental appendix S1.

This case was chosen because anticoagulation therapy is used worldwide, and the issues surrounding the prescription of this medication, patient adherence and especially the temporary discontinuation and resumption of the medication are globally recognised and recurring problems. The top event, defined as the main incident or adverse event that the analysis seeks to understand and prevent in the future and that represents the point at which control was lost, leading to the incident or near-miss,^20^ was presented centrally in advance so that everyone could start formulating recommendations from the same starting point. The top event in this case was defined as ‘anticoagulation treatment has not been adequately resumed’.

Data were gathered in an Excel database; Excel was used for descriptive statistics. The R statistical software environment V.4.3.2 was used for analysis.

**Figure 2:**
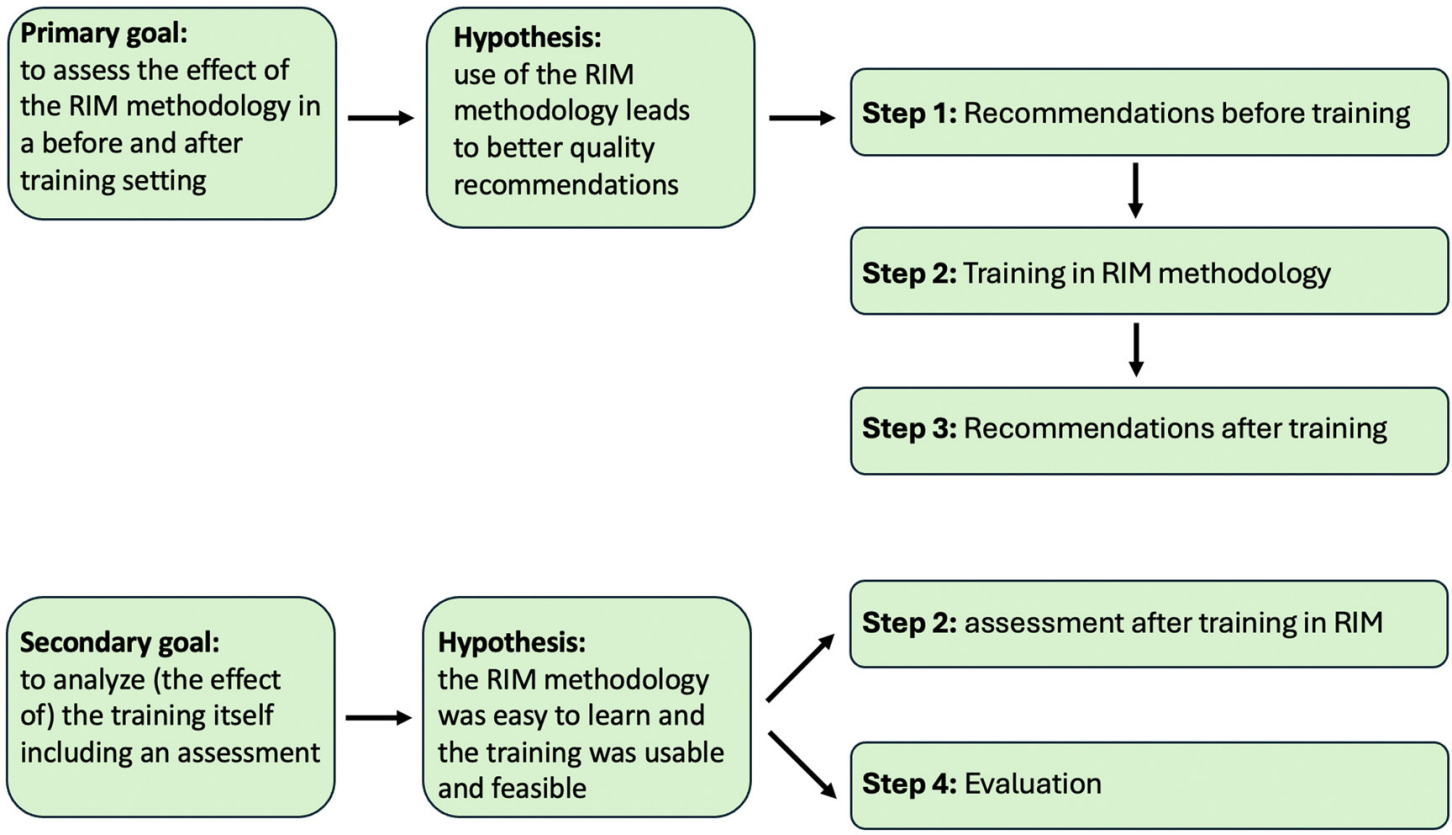
Study design, schematically shown in the research goals, associated hypotheses and research steps to be followed. RIM, recommendation improvement matrix.

The study was conducted in the following steps (figure 2).

### Step 1: recommendations before training

#### Method

After presenting the case, the participants divided into subgroups of a maximum of six people and were asked to draw up recommendations based on the case presented. All recommendations were then assessed through a consensus-based approach by the expert panel. The panel categorised each recommendation as either ‘did pass’ or ‘did not pass’ filter criteria. For those passing the filter criteria, further categorisation was conducted according to the RIM.

#### Data analysis

For each group, all recommendations were recorded and processed anonymously in a database for scientific purposes. The categorisation, as determined by the expert panel, was recorded in both absolute numbers and percentages.

### Step 2: training in RIM methodology and assessment

#### Method

After drawing up the recommendations in the subgroups, all participants were trained in the RIM methodology in class using a different case. The learning objective was that the participants were able to primarily align all their concept recommendations with the three filter criteria^19^ and then to categorise the concept recommendations that passed the filter criteria according to the RIM methodology.^16^ An assessment consisting of eight questions, each with three subquestions: the filter criteria, the category and level of each recommendation to check whether the material had been sufficiently understood. The test questions were reviewed in advance through a consensus-based approach by the expert panel. The panel identified the most appropriate answers so that the answers of the workshop participants could be mirrored.

#### Data analysis

The answer to whether a recommendation passes the filter criteria is displayed dichotomously; the answers for the most appropriate level and category according to the RIM are displayed categorically. For all participants, the test results and individual ratings for the training were processed anonymously in a database for scientific purposes. The test results were presented as the mean (SD) percentage of correct answers per participant. To assess whether there was a difference in test scores across professions, a one-way analysis of variance (ANOVA) was used.

### Step 3: recommendations after training

#### Method

After this assessment, the participants were invited to apply what they had learnt. In the same subgroups, participants discussed among themselves whether the training in the RIM methodology (ie, the filter criteria and the RIM) provided grounds to adjust previously formulated recommendations, formulate additional new recommendations if necessary or remove previously formulated recommendations. In this way, an updated set of recommendations was formulated in each subgroup. All recommendations from all subgroups, both before and after the training, were then consensus-based approaches by the expert panel. The panel categorised each recommendation as either ‘did pass’ or ‘did not pass’ the filter criteria and then categorised them according to the RIM.

#### Data analysis

For each group, all recommendations before and after the training were recorded and processed anonymously in a database for scientific purposes. The quality of the recommendations is expressed in (1) the filter criteria and (2) the level and category according to the RIM. To compare the quality of recommendations before and after the training, the proportion of recommendations that passed the filter was compared with a χ^2^ test.

### Step 4: evaluation

#### Method

At the end of the workshop, participants were asked to rate the feasibility and usability of the new method, using the filter criteria and RIM, on a five-point Likert scale compared with the original method, and to provide explanations and comments about the training.

#### Data analysis

For all participants, the results of the Likert scale and individual remarks were processed anonymously in a database for scientific purposes. Results for feasibility and usability were presented as the average value. A t-test was used for comparisons.

### The research project

This study does not fall under the scope of the Dutch Medical Research Involving Human Subjects Act and therefore does not require approval from an accredited medical ethics committee in the Netherlands. Patients and the public were not involved in the design, conduct or dissemination of this study. Ethical principles were followed, and at the start of the workshop, all participants were briefed on the study aims, data collection process and confidentiality measures. Participation in the workshop was considered as implied informed consent, as attendees voluntarily engaged in the study activities. All participant responses were anonymised and stored securely. Patients and/or the public were not involved in the design, or conduct, or reporting or dissemination plans of this research.

## Results

### Participants

36 professionals participated in the workshop, including eight nurses, four medical doctors, six pharmacologists, four dentists, one general practitioner and six quality officers. The professional background of six participants was not specified. None of the participants had worked with the RIM methodology before. The participants were divided into eight subgroups, each consisting of a maximum of five people. The composition of the subgroups is shown in online supplemental appendix S2.

### Step 1: recommendations before training

All the recommendations made by the subgroups are shown in online supplemental appendix S3. The eight subgroups collectively drew up 3–15 recommendations per group, leading to a total of 49 recommendations prior to the training. Of these 49 recommendations prior to the training, the expert panel classified 18 recommendations (36.7%) as ‘did not meet the filter criteria’ and 31 (63.3%) as ‘did meet the filter criteria’. Of these 31 recommendations, 25 (80.6%) were classified as level B or D, category ‘administrative procedures’ and six as level B, category ‘control’ (19.4%).

### Step 2: training in RIM methodology and assessment

**Figure 3:**
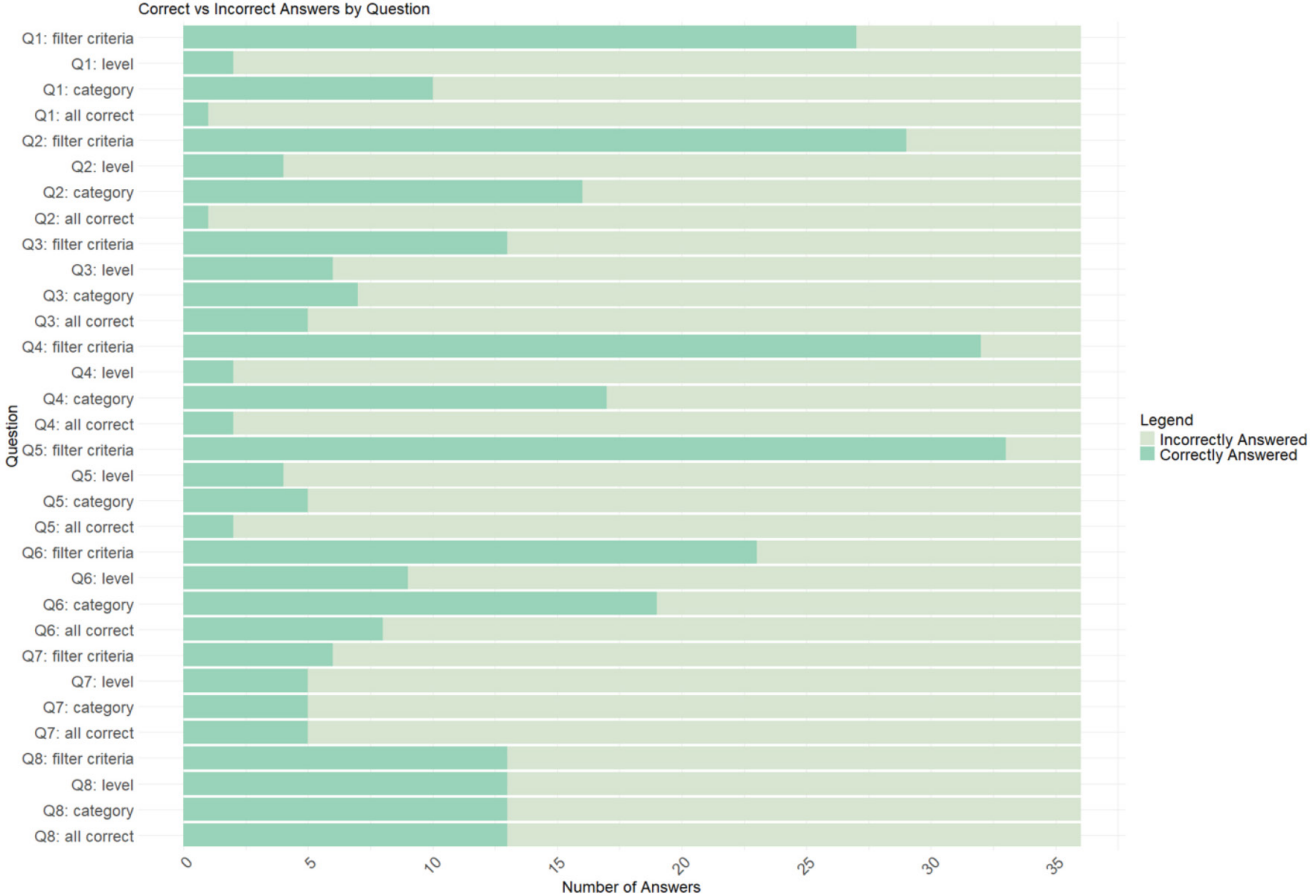
Post-training assessment, distribution of the most appropriate responses.

All 36 participants completed the training in the RIM methodology and subsequently conducted the assessment. Compared with the answers of the expert panel, the participants, on average, answered one of the eight examination questions (13%) correctly. For the subquestions concerning the filter criteria, they answered on average 61% correctly; for the subquestions concerning the appropriate level, 16%; and for the subquestions concerning the correct category, 32% (figure 3 and online supplemental appendix S4). There was no significant difference in the test scores between the different professions (p=0.16, one-way ANOVA).

### Step 3: recommendations after training

Three of the eight subgroups (subgroups 2, 3 and 7, in total 12 participants) did not further participate after the training due to the logistic reasons. Their proposed recommendations (n=10) were excluded from further analysis. The recommendations post-training of the remaining five subgroups totalled 39. In subgroup 4, their last three primary recommendations were not elaborated in the updated set for unclear reasons and were therefore excluded for further analysis (7.7%), bringing the number of recommendations for the before-and-after training analysis to 36.

### Quality of the recommendations

#### Filter criteria

Post-training, the remaining five subgroups excluded 13 (36%) of their 36 original recommendations, leaving 23 (64%) (online supplemental appendix S3).

Considering all 36 recommendations in the before-and-after training analysis, of the 36 recommendations formulated by participants prior to training, 22 (61%) passed the filter according to the expert panel, while 14 (39%) did not.

**Figure 4:**
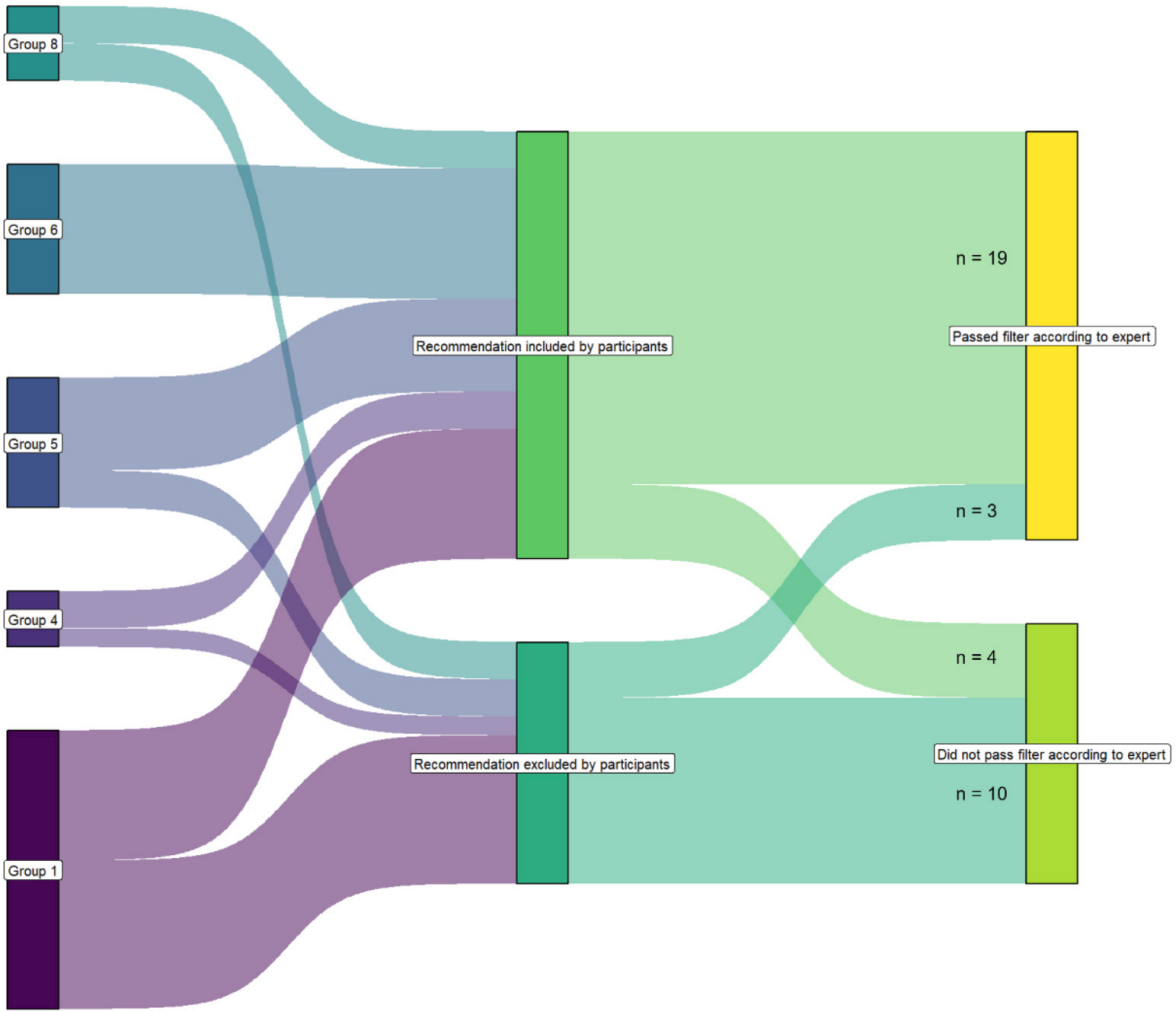
Correct and incorrect use of the filter criteria after training in the recommendation improvement matrix methodology.

The correct and incorrect application of the filter criteria following the training in the RIM methodology is illustrated in figure 4. After the training, 13 recommendations were excluded by the various subgroups. Of these, 10 recommendations (77%) were excluded because they did not meet the filter criteria, as determined by the expert panel.

Comparing the proportion of formulated recommendations that passed the filter according to the expert panel before and after training, it was observed that prior to training, 61% of the recommendations passed the filter, while in the updated set of recommendations after the training, 19 of the 23 (83%) passed the filter. A χ^2^ test was used to compare the proportions, revealing a significant improvement of 22% (p=0.00543).

After training, participants correctly identified 19 of the 22 recommendations as passing the filter, resulting in a sensitivity of 86%. Additionally, they correctly identified 10 of the 14 recommendations as not passing the filter, yielding a specificity of 71%.

#### RIM: level and category

**Figure 5:**
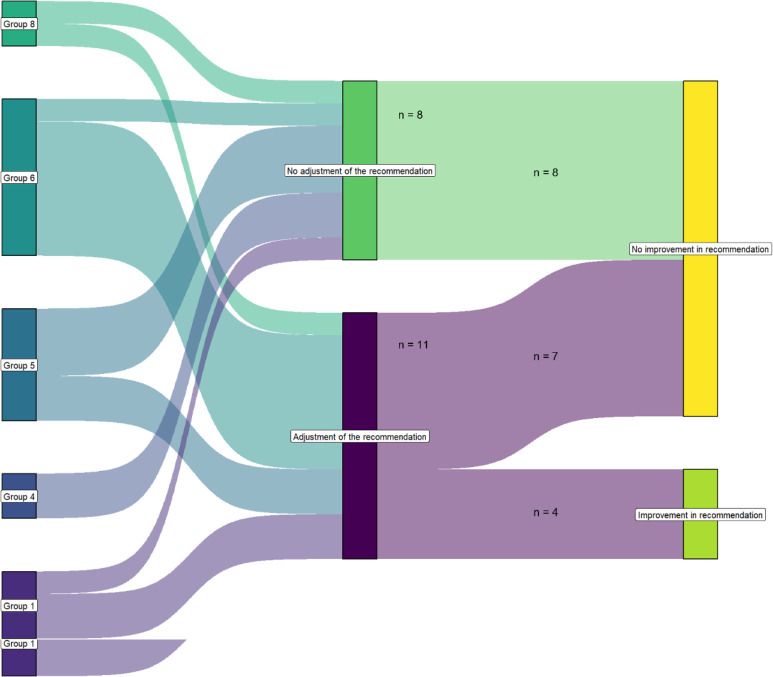
Analysis of before-and-after training in the recommendation improvement matrix methodology regarding the influence of the training on the quality of the recommendations.

After training in the RIM methodology, participants made changes to just over half of the recommendations (11 out of 19), which is illustrated in figure 5. However, this only led to an upgrade in the RIM in a limited number of recommendations. Of the 11 adjusted recommendations, only four (36%) resulted in an upgrade in rating by the expert panel from Level B or D, category ‘administrative procedures’ to Level B or D, category ‘control’. Of the seven recommendations that were not upgraded, six were rated by the expert panel as Level B or D, category ‘administrative procedures’ and one as Level B, category ‘control’. Of the eight recommendations that were not adjusted by the subgroups after the training, the expert panel assessed five as Level B or D, category ‘administrative procedures’ and three as Level B, category ‘control’.

None of the subgroups introduced new recommendations as a result of the training.

### Step 4: evaluation

At the end of the workshop, all participants were asked to rate the feasibility and usability of the RIM methodology on a five-point Likert scale and to provide comments on their ratings. Of the 36 participants, 24 provided responses (see online supplemental appendix S5). On the five-point Likert scale, the average rating for feasibility was 4.39 and for usability was 4.43. Of the 24 participants, 11 (46%) spontaneously indicated a need for further training. These 11 participants were distributed across all subgroups and professional categories. Participants who expressed a need for more training scored higher on the assessment than those who did not. However, when comparing those two groups using a t-test, their test scores were not significantly different (p=0.19). There was also no significant difference between the professions regarding the need for more training (p=0.33, χ^2^ test). Among the largest group of participants, 33% of nurses indicated a need for additional training, yet this group scored the most appropriate answer in the post-training test only 9% of the time.

## Discussion

The analysis of the research results shows an improvement in the formulation of recommendations after training in the RIM methodology. Specifically, the results of this study, conducted during a workshop, demonstrated that professionals trained in the RIM methodology (1) were able to enhance or discard their recommendations after applying the filter criteria and RIM and (2) were able to assess whether their formulated recommendations would effectively address the sentinel event using the RIM. After training, the proportion of recommendations passing the RIM filter increased from 61% to 83% in the paired analysis. These results support our hypothesis that using the RIM methodology leads to better quality recommendations. The results are also in line with our previous findings among inspectors of the Dutch Health and Youth Care Inspectorate and therefore provide additional evidence for the added value of using the RIM methodology.^16^ As far as we know, no other studies have been done that have investigated the use of the RIM methodology.

### Concerning the secondary goal

An analysis of the effect of the training itself through an assessment, in which the responses of individual participants are compared with the answers of the expert panel, the majority of participants found the RIM methodology both feasible and usable. This is also in line with our previous findings among inspectors of the Dutch Health and Youth Care Inspectorate.^16^ However, 46% of the participants indicated that additional training was needed. This finding, spontaneously reported by participants of the workshop, was new to us. We do not recognise this from our previous research but have never actively asked about it. As far as we know, no research has ever been done into the effect of such training itself, and these findings lead to new insights.

## Strengths and limitations

### Strengths

This is the first study in which the same case and the same top event have been used as a starting point by all multidisciplinary study participants to investigate the effects of the RIM methodology on the quality of recommendations. This contrasts with our previous studies, in which each subgroup of participants worked with recommendations from cases they had recently analysed themselves. These recommendations were then reassessed by the participants after training in the RIM methodology to evaluate the effect on the quality of those recommendations. In those studies, each subgroup worked based on its own set of recommendations.

Due to the design of this study, which ensured the same baseline values for each participant, a more robust before-and-after analysis could be conducted. The results indicate that after the training, participants demonstrated a strong ability to correctly identify valid recommendations, as evidenced by a sensitivity of 86%. This suggests that the training effectively enhanced their ability to recognise recommendations meeting the established filter criteria. However, the specificity of 71% indicates that there is room for improvement in accurately identifying recommendations that do not pass the filter. The fact that the participants were multidisciplinary adds to the generalisability of the results.

In previous research, we employed the RIM methodology but did not adequately assess whether participants had truly mastered its use. Our current results indicate that the application of the filter criteria requires only limited training to achieve an improvement in the quality of recommendations. However, proper use of the RIM requires more extensive training. Nonetheless, it appears feasible to further enhance the quality of recommendations with additional training.

At the end of the workshop, all participants were asked to rate, using a five-point Likert scale, the feasibility and usability of the RIM methodology compared with the originally used method. Participants were also invited to explain their scores and provide comments on the training received. Of the 24 participants, 11 (46%) spontaneously indicated a need for additional training. Notably, no comments were made that discredited the quality of the training. This suggests that the training itself was adequate; however, there was insufficient time for participants to fully comprehend the material. Providing more time to practise, particularly with the matrix, appears to offer significant added value.

The need for additional training is further supported by the post-training test results, which revealed that only 13% of participants provided the most appropriate answers as determined by the expert panel. In our previous studies, we did not explicitly test whether the training was adequately understood. This aspect warrants attention in future research. It is plausible that enhanced training, coupled with the implementation of a robust assessment framework to evaluate comprehension of the material, could further amplify the benefits of the RIM methodology.

Also noteworthy in this context is the relationship between the expressed need for additional training and the post-training test results among the group of nurses. Although the group consisted of only six responding participants, only two nurses (33%) indicated a need for more training, despite this group achieving the most appropriate answers in the post-training test in only 9% of cases. This discrepancy suggests a potential gap between the perceived need for further training and the actual requirement based on performance. These findings support the importance of implementing a robust assessment framework to objectively evaluate comprehension of the material.

The evident need for additional training warrants careful consideration in future research and should be accounted for in the interpretation of the findings of this study. Enhanced and more comprehensive training has the potential to further improve the formulation of optimal recommendations. A recommended approach to achieving this could involve incorporating practice with multiple cases, accompanied by focused discussions. Training should prioritise the assessment and application of the RIM, while placing comparatively less emphasis on the filter criteria, which appear to require less extensive instruction.

### Limitations

The limited number of participants in this study is undoubtedly a key limitation, which may have contributed to the absence of statistically significant results in some analyses. Moreover, the setting, being a conference in which this study was embedded, might have influenced the results. Although the time frame for the session was clear, participants were able to attend as they wished. Another possible limitation is the presented case. The case might not have been relevant to all participants. It would be impossible to present a case relevant to all participants. By taking the time and elaborately presenting the case, the team tried to mitigate this issue.

Following the training in the RIM methodology, participants changed just over half of their recommendations, indicating that the methodology may have an impact. However, this did not lead to a significant improvement in the level or category of the recommendation as assessed by the RIM. It remains unclear whether this lack of significant upgrades is due to insufficient training on these aspects or because the matrix itself does not inherently facilitate substantial improvement. Further research would be needed to clarify this point. Since recommendations for improving safety to prevent a sentinel event are usually made after an in-depth analysis, it is also debatable to what extent the participants are able to make relevant recommendations at all after reading the brief description of the case and under time pressure.

The observed request and perceived need for additional training was a new phenomenon in this study. In previous studies, we made the general supposition that the RIM methodology could be learnt easily and in a short time.

A potential explanation for the discrepancy between earlier experiences and the current findings could be the diverse professional backgrounds of the participants. In addition to medical doctors and quality officers, the participant pool included nurses, pharmacists and dentists. In our prior research, participants were exclusively medical doctors and/or quality officers.

The case study employed in this workshop was based on a hospital setting, which may have influenced the results. Dentists, in particular, may have been less familiar with the pathology described in the case, potentially affecting their engagement and performance during the training. Another explanation for this discrepancy between previous experiences and the current outcome could be that the training and the test were offered in English, while for the participants as well as the trainers, this was not their native language.

### Recommendations

We recommend extending the training to more than 1 day, piloting the revised format and comparing it to the current structure to measure usability and impact. This additional time would enable all participants to develop a shared understanding of the material. A longer duration would allow for comprehensive coverage of key topics, interactive discussions and hands-on practice, ensuring that participants with varying levels of prior knowledge can effectively grasp the concepts.

Such an extended format would foster a more inclusive learning environment and enhance the overall impact of the workshop. Furthermore, we suggest incorporating this workshop into the medical curriculum or offering it as a highly recommended continuing professional development programme. This approach would help ensure that healthcare professionals are equipped with the necessary skills and knowledge to keep pace with advancements in the field.

## Conclusion

Analysis of the research results shows an improvement in the formulation of recommendations following training in the RIM methodology.

Specifically, the findings from this workshop indicate that professionals trained in the RIM methodology were able to (1) evaluate whether their formulated recommendations were structured in a way that would effectively address the sentinel event using the RIM methodology’s filter criteria and (2) enhance or discard recommendations as needed based on the RIM methodology.

The majority of participants found the RIM methodology both feasible and usable. However, a significant proportion also expressed the need for additional training. While the training successfully improved participants' ability to identify valid recommendations, further efforts are necessary to enhance their capability to accurately identify and address non-compliant recommendations. This would help minimise the inclusion of potentially weak recommendations in future assessments.

It is plausible that with more comprehensive training, particularly with a stronger emphasis on the RIM, the ability to formulate optimal recommendations could be further improved, amplifying the overall effectiveness of the RIM methodology. Future research could be a cluster-randomised or stepped-wedge study comparing RIM training with usual practice. This research should examine learning curves, retention and training dose-response. Psychometric evaluation of the matrix is needed to improve reliability and validity. And most importantly, linking RIM quality to implementation success and patient safety outcomes is essential.

## Supplementary material

10.1136/bmjopen-2025-101743 online supplemental file 1

## Data Availability

Data are available upon reasonable request. All data relevant to the study are included in the article or uploaded as supplementary information.
