# Supplementary material for: Improved quality of recommendations after sentinel event analysis with recommendation improvement matrix training: a before-and-after study at an international patient safety conference
Source: BMJ Open. 2025 Nov 12;15(11):e101743. doi: 10.1136/bmjopen-2025-101743 (PMC12612720; doi:10.1136/bmjopen-2025-101743)
Supplement: online supplemental file 1 [file bmjopen-15-11-s001.docx]

**Supplemental material**

**Appendix 1** Case presentation workshop

**Workshop Adverse Events Recommendation Improvement Matrix**

Analyses of adverse events could it be otherwise, better?

**Peter de Feiter, Dave Dongelmans**, **Annelies Visser**

4th SPSC International Patient Safety Conference Saudi Arabia

**Case presentation**

The case, really happened in 2022

A 69-year-old patient visits the Urology outpatient clinic because of a kidney stone with hydronephrosis. An indication for drainage is set.

His medical history mentions an aortic valve insufficiency and mitral valve insufficiency, for which aortic valve replacement and mitral valveplasty and a pacemaker related to asystoles under beta blocker (needed to control atrial fibrillation).

As anticoagulation, he uses a coumarin derivative.

Patient is being treated with chemoradiation in another hospital during the same period for a nasopharyngeal carcinoma recently diagnosed abroad. The kidney stone and hydronephrosis were discovered by chance on the PET scan made abroad because of nosebleeds in relation to the nasopharyngeal carcinoma.

Verbal arrangements were made with the patient about his anticoagulation prior to the procedure: three days prior to the placement of the nephrostomy drain, he must stop taking the coumarin and if he no longer urinates bloody after the procedure, he can resume the anticoagulation 24 hours later. Written information is not provided; It is also not available in the hospital. The patient has been arranging his anticoagulation himself for many years in consultation with the thrombosis service and this service was also aware of the temporary cessation of the coumarin.

Prior to the procedure, a control INR was done and the value of this was good for the placement of the nephrostomy drain. Installation of the nephrostomy drain took place on 27-07-2022. After nephrostomy drain placement, patient was discharged home after a short observation period of several hours. At the time of patient discharge, there was no further discussion of the conditions under which the patient could resume his anticoagulation, nor was there any mention of this in discharge status, discharge letter or patient information that patients can review at home.

On 11-08-2022, the patient unfortunately develops a Cerebrovascular Accident with severe symptoms. It appeared that he did not restart with the coumarin, because he read in his file at home after the procedure under his medication overview for coumarin: 'Starting from August 20' a restart schedule was also added. The patient was also in possession of a card with a start-up schedule from the thrombosis service but had read on it: 'Unless the doctor decides otherwise'. The thrombosis service never verifies this with the doctors involved.

Upon further investigation by the pharmacy, it appeared that this was a medication order from August *2020* during admission due to a subdural hematoma in which the coumarin had been temporarily discontinued. The doctor who prescribed discharge medication at that time placed the relevant restart schedule in the free text of the prescription. The free text space of the prescription for use is not intended for this and therefore inadvertently comes back every time (with every Hospitalization). The free text of the prescription for use is intended for a note that is necessary to read with *each* repeat prescription of a medicine. The restart schedule noted here cannot be seen during medication verification, but the patient does see (indefinitely) in his personal file.

You are requested to analyze this sentinel event.

As top event, you choose the definition:

'Anticoagulation treatment has not been adequately resumed.'

Now formulate recommendations based on the case and the defined top event in subgroups according to the methodology you usually use and write down these completely written recommendations.

**Appendix 2** Distribution of professionals among the subgroups

| subgroup | nurse | medical doctor | pharmacist | dentist | general practitioner | quality officer | unknown | total |
| --- | --- | --- | --- | --- | --- | --- | --- | --- |
| 1 |  | 1 | 1 | 2 |  | 1 |  | 5 |
| 2 |  | 1 | 1 | 1 |  | 1 |  | 4 |
| 3 | 1 | 1 |  |  |  |  | 2 | 4 |
| 4 | 1 |  | 3 |  |  | 1 |  | 5 |
| 5 | 1 | 1 |  |  |  | 2 | 1 | 5 |
| 6 |  |  | 1 | 1 |  |  | 2 | 4 |
| 7 | 2 |  |  |  |  |  | 2 | 4 |
| 8 | 3 |  |  |  | 1 | 1 |  | 5 |

**Appendix 3** Recommendations made by the subgroups before and after training.

Expert Panel Assessment Legend:

does not pass filter criteria : 0

level conform RIM methodology : A t/m D

category: Elimination : 1

Substitution : 2

Control : 3

Administrative procedures : 4

category personal protection : 5

changed after training : / c

unchanged after training : / u

| **Subgroup 1** | **Recommendations** | **Assessment by expert panel / text (un)changed** |
| --- | --- | --- |
| **before training** | 1. take two opinions from two clinics | 0 |
|  | 2. make verbal agreement with patient | B4 |
|  | 3. write investigation in document | B4 |
|  | 4. time of discharge ….. was no attention about anticoagulant | 0 |
|  | 5. patient was not concern about medication after surgery | 0 |
|  | 6. the team must be known their responsibility ((administrative) | 0 |
|  | 7. must be written in digital desk (substitution) | 0 |
|  | 8. patient education about the medication (control) | B3 |
|  | 9. multi-disciplinary team (it's same no 1) | 0 |
|  | 10. developing computer provider order system | B3 |
|  | 11. update patient state (administrative) | 0 |
|  | 12. post operative instruction written document (administrative) | B4 |
|  | 13. enhancing communication between physician and patient (personal protection) | 0 |
|  | 14. follow up schedule | 0 |
|  | 15. continue education for the whole team | 0 |
| **after training** | 8. cleary the medication for the patient and educate him | B3 / c |
|  | 10. develop a computer order system | B3 / u |
|  | 11. updating patient state always | 0 |
|  | 12. postoperative instruction must be given to patient in written form | B4 / c |
|  | 13. enhancing communication between doctors and patients | 0 |
|  | 14. follow up schedule | 0 |
|  | 15. continue education for whole team working in hospital | 0 |
|  |  |  |
| **Subgroup 2** | **Recommendations** | **Assessment by expert panel** |
| **before training** | 1. initiation of unified (national) medical record (B control) | 0 |
|  | 2. involve care giver in treatment plans and management (B control) | 0 |
|  | 3. write patient information post admission and procedure | B4 |
|  | 4. make a time limit o free text pharmacy instruction and update every time of refill | B4 |
| **after training** | none |  |
|  |  |  |
| **Subgroup 3** | **Recommendations** | **Assessment by expert panel** |
| **before training** | 1. perform thorough medication review during admission to ensure complete medication history | 0 |
|  | 2. encourage better medical documentation for healthcare team to improve communication of patient data | B4 |
|  | 3. create a list to establish a systematic pathway for discharge planning ( utilize patient family health education; outpatient referrals (follow ups) to appropriate facilities) | B3 |
| **after training** | none |  |
|  |  |  |
| **Subgroup 4** | **Recommendations** | **Assessment by expert panel / text (un)changed** |
| **before training** | 1. root cause analysis for all possible causes to address the findings | 0 |
|  | 2. to establish a referral system for proper communication and sharing full patient information and history | B4 |
|  | 3. Assure proper reconciliation at admission, transfer, during procedure and for discharge | B3 |
|  | 4. review the discharge planning, process and outcome and close the gaps | D4 |
|  | 5. Improve the documentation process among the healthcare management (reconciliation verbal agreement, prescribing medication and the free text of R/x) | B3 |
|  | 6. Develop/modify the discharge policy | D4 |
| **after training** | 2. to establish a referral system for proper communication and sharing full patient information and history | B4 / u |
|  | 3. Assure proper reconciliation at admission, transfer, during procedure and for discharge | B3 / u |
|  |  |  |
| **Subgroup 5** | **Recommendations** | **Assessment by expert panel / text (un)changed** |
| **before training** | 1. National health system for shared electronic info (accessibility of patient history) through / include referral system (policy) | D4 |
|  | 2. Ensure proper documentation (medical & status & medication etc.) through auditing | D4 |
|  | 3. Elimination of system / electronic errors by filling of mandatory fields by drop lists instead of free text | D4 |
|  | 4. medication reconciliation | D4 |
|  | 5. Training & education of all staff for proper documentation & following the guidelines and pathways | D4 |
|  | 6. Improve staff communication | 0 |
|  | 7. Patient education & engagement | 0 |
| **after training** | 1. Create National database for patients’ health electronic files | D4 / c |
|  | 2. Establish an electronic pharmacy system to ensure appropriate completed medication | D3 / c |
|  | 3. Elimination of system / electronic errors by filling of mandatory fields by drop lists instead of free text | D4 / u |
|  | 4. medication reconciliation | D4 / u |
|  | 5. Training & education of all staff for proper documentation & following the guidelines and pathways | D4 / u |
|  |  |  |
| **Subgroup 6** | **Recommendations** | **Assessment by expert panel / text (un)changed** |
| **before training** | 1. written instruction about anticoagulation pre-/post-surgical | D4 |
|  | 2. forcing function to prevent wrong … (prescription) | B4 |
|  | 3. patient medication education prior to discharge | D4 |
|  | 4. system design to highlight and arrange order in chronological order | D4 |
|  | 5. discharge plan -> including follow up… to return to clinic | B4 |
|  | 6. medication reconciliation and involve clinical pharmacists | D4 |
|  | 7. reporting of the incident and using as opportunity for improvement | D4 |
| **after training** | 1. written instructions about anticoagulation for patient prior surgical / post-surgical | D4 / c |
|  | 2. software installment to force data entry to support decision for clinicians | B3 / c |
|  | 3. patient / family education about care plan prior to discharge | B3 / c |
|  | 4. system design to highlight arranging orders in chronological order | D4 / u |
|  | 5. design a process to coordinate patient follow up appointments with primary clinic specially patients with comorbidities | B4 / c |
|  | 6. design a process for medication reconciliation before discharge | B3 / c |
|  | 7. activate incidence reporting, just culture, use these incidences as opportunity for improvement | D4 / c |
|  |  |  |
| **Subgroup 7** | **Recommendations** | **Assessment by expert panel** |
| **before training** | 1. preprocedure patient education -> written clear instructions | 0 |
|  | 2. discharge (post procedure) discharge instructions | D4 |
|  | 3. medication management -> 2-layer checking: a: prescription (physician), b: dispense (pharmacist) | D4 |
| **after training** | none |  |
|  |  |  |
| **Subgroup 8** | **Recommendations** | **Assessment by expert panel / text (un)changed** |
| **before training** | 1. to improve communication with multidisciplines of care MRP … thrombosis team | 0 |
|  | 2. there should be a clear discharge summary to be given to patient by written information, especially instructions for medications | D4 |
|  | 3. Hospital information system improvement to add one-step in free text prescription to be verified by the pharmacist | B3 |
|  | 4. Full compliance on post-OP care, follow-up by the surgery dep. and VTE pp | D4 |
| **after training** | 3. Improvement of hospital information system, to add one more step in free text prescription, to be verified by the pharmacy | B3 / u |
|  | 4. Full compliance on policies, procedures and auditing (policy proc. Documentation, policy proc. Discharge, policy proc. VTE, policy proc. Pt. Assessment / re-assessment | D4 / c |

**Appendix 4** Post-training assessment. Distribution of the most appropriate responses among the subgroups and the different professional backgrounds (number/total (%))

| test question | correct answer | nurse | medical doctor | pharmacist | dentist | general prctitioner | quality officer | unknown | total |
| --- | --- | --- | --- | --- | --- | --- | --- | --- | --- |
| 1 | filter criteria | 8/8 | 3/4 | 4/6 | 2/4 | 1/1 | 4/6 | 5/7 | 27/36 (75%) |
|  | level | 0/8 | 0/4 | 0/6 | 0/4 | 0/1 | 1/6 | 1/7 | 2/36 (6%) |
|  | category | 1/8 | 2/4 | 2/6 | 2/4 | 0/1 | 1/6 | 2/7 | 10/36 (28%) |
|  | all correct | 0/8 | 0/4 | 0/6 | 0/4 | 0/1 | 0/6 | 1/7 | 1/36 (3%) |
| 2 | filter criteria | 8/8 | 3/4 | 4/6 | 4/4 | 0/1 | 5/6 | 5/7 | 29/36 (81%) |
|  | level | 3/8 | 0/4 | 1/6 | 0/4 | 0/1 | 0/6 | 0/7 | 4/36 (11%) |
|  | category | 3/8 | 2/4 | 3/6 | 2/4 | 0/1 | 3/6 | 3/7 | 16/36 (44%) |
|  | all correct | 0/8 | 0/4 | 1/6 | 0/4 | 0/1 | 0/6 | 0/7 | 1/36 (3%) |
| 3 | filter criteria | 4/8 | 1/4 | 1/6 | 0/4 | 0/1 | 3/6 | 4/7 | 13/36 (36%) |
|  | level | 2/8 | 0/4 | 1/6 | 0/4 | 0/1 | 1/6 | 2/7 | 6/36 (17%) |
|  | category | 1/8 | 1/4 | 1/6 | 0/4 | 0/1 | 1/6 | 3/7 | 7/36 (19%) |
|  | all correct | 1/8 | 0/4 | 1/6 | 0/4 | 0/1 | 1/6 | 2/7 | 5/36 (14%) |
| 4 | filter criteria | 8/8 | 3/4 | 5/6 | 4/4 | 1/1 | 5/6 | 6/7 | 32/36 (89%) |
|  | level | 0/8 | 0/4 | 1/6 | 1/4 | 0/1 | 0/6 | 0/7 | 2/36 (6%) |
|  | category | 5/8 | 2/4 | 2/6 | 4/4 | 0/1 | 2/6 | 2/7 | 17/36 (47%) |
|  | all correct | 0/8 | 0/4 | 1/6 | 1/4 | 0/1 | 0/6 | 0/7 | 2/36 (6%) |
| 5 | filter criteria | 8/8 | 4/4 | 5/6 | 4/4 | 0/1 | 5/6 | 7/7 | 33/36 (92%) |
|  | level | 0/8 | 0/4 | 1/6 | 2/4 | 0/1 | 1/6 | 0/7 | 4/36 (11%) |
|  | category | 0/8 | 1/4 | 1/6 | 0/4 | 0/1 | 1/6 | 2/7 | 5/36 (14%) |
|  | all correct | 0/8 | 0/4 | 1/6 | 0/4 | 0/1 | 1/6 | 0/7 | 2/36 (6%) |
| 6 | filter criteria | 5/8 | 3/4 | 2/6 | 2/4 | 0/1 | 4/6 | 7/7 | 23/36 (64%) |
|  | level | 1/8 | 1/4 | 1/6 | 1/4 | 0/1 | 1/6 | 4/7 | 9/36 (25%) |
|  | category | 4/8 | 3/4 | 0/6 | 2/4 | 0/1 | 3/6 | 7/7 | 19/36 (53%) |
|  | all correct | 1/8 | 1/4 | 0/6 | 1/4 | 0/1 | 1/6 | 4/7 | 8/36 (22%) |
| 7 | filter criteria | 0/8 | 3/4 | 1/6 | 0/4 | 0/1 | 0/6 | 2/7 | 6/36 (17%) |
|  | level | 0/8 | 2/4 | 1/6 | 0/4 | 0/1 | 0/6 | 2/7 | 5/36 (14%) |
|  | category | 0/8 | 2/4 | 1/6 | 0/4 | 0/1 | 0/6 | 2/7 | 5/36 (14%) |
|  | all correct | 0/8 | 2/4 | 1/6 | 0/4 | 0/1 | 0/6 | 2/7 | 5/36 (14%) |
| 8 | filter criteria | 4/8 | 2/4 | 4/6 | 1/4 | 0/1 | 0/6 | 2/7 | 13/36 (36%) |
|  | level | 4/8 | 2/4 | 4/6 | 1/4 | 0/1 | 0/6 | 2/7 | 13/36 (36%) |
|  | category | 4/8 | 2/4 | 4/6 | 1/4 | 0/1 | 0/6 | 2/7 | 13/36 (36%) |
|  | all correct | 4/8 | 2/4 | 4/6 | 1/4 | 0/1 | 0/6 | 2/7 | 13/36 (36%) |
| total results | filter criteria | 45/64 (70%) | 22/32 (69%) | 26/48 (54%) | 17/32(53%) | 2/8 (25%) | 26/48 (54%) | 38/56 (68%) | 176/288 (61%) |
|  | level | 10/64 (16%) | 5/32 (16%) | 10/48 (21%) | 5/32 (16%) | 0/8 (0%) | 4/48 (8%) | 11/56 (20%) | 45/288 (16%) |
|  | category | 18/64 (28%) | 15/32 (47%) | 14/48 (29%) | 11/32 (34%) | 0/8 (0%) | 11/48 (23%) | 23/56 (41%) | 92/288 (32%) |
|  | all correct | 6/64 (9%) | 5/32 (16%) | 9/48 (19%) | 3/32 (9%) | 0/8 (0%) | 3/48 (6%) | 11/56 (20%) | 37/288 (13%) |

**Appendix 5** Evaluation: survey of feasibility and usability among workshop participants.

Legend:

Profession: nurse : 1

medical doctor : 2

pharmacist : 3

dentist : 4

general practitioner : 5

quality officer : 6

Feasibility and usability on 5-point Likert-scale

Need more training: yes : 1

no : 0

missing data : ?

| **subgroup** | **profession** | **feasibility** | **explain score** | **usability** | **explain score** | **need more training** |
| --- | --- | --- | --- | --- | --- | --- |
| 1 | 2 | 5 | It will improve outcome | 5 | Will …. Explained | 0 |
| 1 | 4 | 4 | think the presentation need more sample to get really use to the RIM but thank you so much for the whole information | 4 | need to use more example and more explain to know the whole different | 1 |
| 1 | 4 | 4 | It is a good evaluation method, but the level is not clear | 4 | it could be used, but need more explanation | 1 |
| 1 | 3 | 4 | It helps mainly to prioritize the risk and identify it with it's impact | 4 | It's easy to use it and will open the possibilities t choose the best recommendations sometimes there will be some difference opinions when assess the recommendations but it's helpful | 0 |
| 1 | 6 | 4 | If there is more time to solve cases and have integration between all the participate will be more effect | 5 | It's a new concept, need time to implement it well in the institution | 1 |
| 2 | 4 | 4 | It is a good method to guide us but need more clarification and different people have different opinions on score wise | 4 | it can be used even though people have different views to scale situations, and it should be more descriptive | 1 |
| 2 | 6 | 3 | The framework of RIM is … However, it needs to be explained in a large amount of time to understand it fully before using it. M table team found a hard time understanding the boxes and their associated terms. | 4 | I believe that the framework will work if it is explained in an depth and series of lectures. This framework is supposed to be taught in a 3-4 weeks to fully understand it. | 1 |
| 2 | 2 | 4 | The RIM set up critics to any recommendation and set up a prioritizing method | 3 | need more training and orientation to clear some ambiguity … it has | 1 |
| 2 | 3 | 4 | It was organized in a nice way; however we need to differentiate between … from the left side about the subcategories | 5 | clear and measurable | 0 |
| 3 | 1 | 5 | The filter criteria and RIM will help us determine the appropriate recommendations. This will save valuable time and recourses | 5 | The filter criteria and RIM need to be taught clearly for the concerned persons to be able fully maximize its use. | 1 |
| 3 | 2 | ? | ? | ? | ? | ? |
| 3 | ? | 5 | It is very informative | 5 | ? | ? |
| 3 | ? | 5 | For me it gives me idea to improvement sentinel events from reoccurring in the future that force us … protocol/method to resolve the problem | 5 | it is usable to recognize the problem | 0 |
| 4 | 6 | 5 | It is not about he recommendation, it is how to implement it and how to improve the outcomes. | 5 | To find out the clearance and usability of the recommendations | 0 |
| 4 | 3 | 5 | Categorization helped in assessing the recommendation and guided in to decide whether it is reliable or not | 4 | It is somehow easy, but needs more practice (some confusion) | 1 |
| 4 | 3 | 5 | ? | 5 | ? | ? |
| 4 | 3 | 4 | ? | 5 | ? | ? |
| 4 | 1 | 5 | ? | 5 | This matrix is a valuable in which made me think of each recommendation and filter then before approval | 0 |
| 5 | ? | ? | ? | ? | ? | ? |
| 5 | 6 | ? | ? | ? | ? | ? |
| 5 | 6 | ? | ? | ? | ? | ? |
| 5 | 2 | 5 | This method filters the recommendations provide and narrow toward to the most strong and proper ones | 4 | just because not all are aware about this methods and need to practice more and educate them about it. | 1 |
| 5 | 1 | 4 | ? | 5 | I think it is great as it is guide me to think critically before I suggest a recommendation and to select the best of them | 0 |
| 6 | 4 | 5 | ? | 4 | Just not sure how can intervention happen before S.E. be written in the recommendation because usually it is not in our facilities. | 0 |
| 6 | 3 | 5 | ? | 5 | Is easy to use but I want to know how reach level 1 without reach harmful …. | 0 |
| 6 | ? | ? | ? | ? | ? | ? |
| 6 | ? | ? | ? | ? | ? | ? |
| 7 | 1 | ? | ? | ? | ? | ? |
| 7 | ? | 4 | ? | 4 | ? | ? |
| 7 | 1 | ? | ? | ? | ? | ? |
| 7 | ? | 4 | The third point in the filter is not really applicable in evaluation of individual cases | 4 | for the same reason criterion no 3 needs to be revised or rephrased | 0 |
| 8 | 6 | 4 | It is a helpful system in a reactive response, but it was unclear a little | 5 | It is more systematic and wider ranged | 1 |
| 8 | 1 | 5 | Is a very new method to me. I found it real valuable, but I guess I need more exercises to be familiar | 4 | will be using this mor frequent to be familiar | 1 |
| 8 | 1 | 4 | It is easy to understand and easy for learn some methods | 4 | it is easy workshop | 0 |
| 8 | 1 | 4 | It is easy guide us to select the solution, easy understanding | 4 | easy to use and to be understanding the selection the solution | 0 |
| 8 | 5 | 4 | It is easy to understand, easy to select and protocol-based method | 4 | easy approach | 0 |
