# Supplementary material for: Improved quality of recommendations after sentinel event analysis with recommendation improvement matrix training: a before-and-after study at an international patient safety conference
Source: BMJ Open. 2025 Nov 12;15(11):e101743. doi: 10.1136/bmjopen-2025-101743 (PMC12612720; doi:10.1136/bmjopen-2025-101743)
Supplement: Supplementary file 2 [file bmjopen-2025-101743.reviewer_comments.pdf]

PEER REVIEW HISTORY

BMJ Open publishes all reviews undertaken for accepted manuscripts. Reviewers are asked to complete a checklist review form and are provided with free text boxes to elaborate on their assessment. These free text comments are reproduced below.

ARTICLE DETAILS

Title (Provisional)

Improved quality of recommendations after sentinel event analysis with Recommendation Improvement Matrix training: A before-and-after study at an international patient safety conference

Authors

Feiter, Peter de; Visser, Annelies; Al Baharnah, Alia; Alkutbe, Rabab; Bakker, Tinka; Asery, Ali; Dongelmans, Dave

---

VERSION 1 - REVIEW

**Reviewer**	**1**
**Name**	**Polluste, Kaja**
**Affiliation**	**University of Tartu, Department of internal Medicine**
**Date**	**04-Apr-2025**
**COI**	**None**

The aim of this paper is to evaluate the effectiveness of the Recommendation Improvement Matrix (RIM) methodology in improving the quality of recommendations after a sentinel event analysis. The authors hypothesize that the use of the RIM methodology leads to better quality recommendations. By evaluating the quality of recommendations before and after a workshop in which participants were trained to use the RIM methodology, the authors concluded that the RIM methodology significantly improves the quality of recommendations following sentinel event analysis.

1) Abstract

In the abstract, the wording of Design and Intervention could be improved.

The current wording of the Intervention "Participants formulated recommendations on the sentinel event case before and after receiving training in the RIM methodology" is a little difficult to understand. It could be interpreted that the Intervention is a formulation of recommendations. However, the Intervention is a RIM training and the quality of the recommendations formulated is the indicator against which the effectiveness of the

intervention is assessed. Thus, I would suggest formulating the Intervention simply as RIM methodology training during a structured 3-hour workshop.

The quality of the formulated recommendations could be included in the wording of Design, for example as follows: a before-and-after analysis of the quality of the formulated recommendation after the sentinel event analysis.

2) Methodology

The study design is explained but it's rather hard to follow. There are two parts of the research project – is the research project the current study? Additionally, the authors describe four steps of the workshop. It's not clear how the two parts of the research project are linked to the four steps of the workshop.

I would suggest to present the study design as a figure where all parts and steps and connection between them could be visualized, and add the main points of both parts and all four steps on the figure. Using the figure the study design could be more clear and better understandable.

The design of the survey has been explained, but it is quite difficult to follow. There are two parts of the research project - is the research project the current study? In addition, the authors describe the four stages of the workshop. It is not clear how these two parts of the research project relate to the four stages of the workshop.

I would suggest presenting the research project as a diagram (e.g. a flowchart), where all the parts and steps and the relationship between them can be visualized, and including in the diagram the main points of both parts and all four steps. The figure would make the study design clearer and easier to understand, and would also allow the reader to better understand the results.

3) Discussion

The discussion is short, mainly repeats the results and focuses on describing the strengths and weaknesses of the work. There is no discussion of the results in relation to those published in previous studies. The discussion does not refer to any references. As recommendations for improving safety to prevent a sentinel event are usually made after an in-depth analysis, the question remains to what extent the participants are in a position to make relevant recommendations at all after having read the brief description of the case. This could be discussed as a limitation, too.

4) References

Eight references have been cited in the writing of this article, one of which has been submitted for publication but not yet accepted.

Minor comment:

The colour scheme used in Figure 2 could be revised. The two intense colours make it difficult to follow the figure. For example, a darker and lighter shade of one colour could be used.

**Reviewer**	**2**
**Name**	**López-Hernández , Luz Berenice**
**Affiliation**	**Universidad Autónoma de Guadalajara, Ciclo de Vida**
**Date**	**26-Jul-2025**
**COI**	**None**

The authors provide a thought-provoking analysis of a critical gap in sentinel event recommendations—an issue with significant implications for patient safety and healthcare quality. Although the sample size is limited, the study offers meaningful contributions to the field.

However, the introduction is too brief and lacks sufficient context, which may obscure the study's rationale. Expanding this section to clearly outline the background, significance, and objectives would strengthen the manuscript.

Additionally, the discussion could delve deeper into how the findings compare with existing strategies and should emphasize key takeaways that align with the study's goals. Currently, the document feels unbalanced: the introduction and discussion are too short, while other sections are overly descriptive, disrupting the flow. A more structured and proportionate presentation would improve readability and impact.

**** RIM review.pdf ****

VERSION 1 - AUTHOR RESPONSE

Reviewer 1 Comments
Dr. Kaja Polluste, University of Tartu
Comments to the Author:
The aim of this paper is to evaluate the effectiveness of the Recommendation Improvement Matrix (RIM) methodology in improving the quality of recommendations after a sentinel event analysis. The authors hypothesize that the use of the RIM methodology leads to better quality recommendations. By evaluating the quality of recommendations before and after a workshop in which participants were trained to use the RIM methodology, the authors concluded that the RIM methodology significantly improves the quality of recommendations following sentinel event analysis.

Comment 1: Abstract
In the abstract, the wording of Design and Intervention could be improved.
The current wording of the Intervention "Participants formulated recommendations on the sentinel event case

before and after receiving training in the RIM methodology" is a little difficult to understand. It could be interpreted that the Intervention is a formulation of recommendations. However, the Intervention is a RIM training and the quality of the recommendations formulated is the indicator against which the effectiveness of the intervention is assessed. Thus, I would suggest formulating the Intervention simply as RIM methodology training during a structured 3-hour workshop.
The quality of the formulated recommendations could be included in the wording of Design, for example as follows: a before-and-after analysis of the quality of the formulated recommendation after the sentinel event analysis

Reply 1:
We sincerely thank the reviewer for her valuable time, effort, and constructive feedback. We do agree that the wording of Design and Intervention could be improved and we have adjusted Design and Interventions in the abstract in accordance with the reviewer's suggestions.

Changes in the text:
Design: A before-and-after analysis of the quality of the formulated recommendations after sentinel event analysis.
Interventions: RIM methodology training during a structured 3-hour workshop.

Comment 2: Methodology
The study design is explained but it’s rather hard to follow. There are two parts of the research project – is the research project the current study? Additionally, the authors describe four steps of the workshop. It’s not clear how the two parts of the research project are linked to the four steps of the workshop.
I would suggest to present the study design as a figure where all parts and steps and connection between them could be visualized, and add the main points of both parts and all four steps on the figure. Using the figure the study design could be more clear and better understandable.
The design of the survey has been explained, but it is quite difficult to follow. There are two parts of the research project - is the research project the current study? In addition, the authors describe the four stages of the workshop. It is not clear how these two parts of the research project relate to the four stages of the workshop.
I would suggest presenting the research project as a diagram (e.g. a flowchart), where all the parts and steps and the relationship between them can be visualized, and including in the diagram the main points of both parts and all four steps. The figure would make the study design clearer and easier to understand, and would also allow the reader to better understand the results.

Reply 2:
We thank the reviewer for her valuable time, and constructive feedback, and strongly agree with this comment. As suggested by the reviewer, we have added a flowchart (figure 2) to the text and adjusted the text itself:

1. We moved the paragraph in which we explain the RIM methodology to the introduction section (see also comment 1 by reviewer 2).
2. We moved the paragraph in which we explain the ethical aspects called ‘the research project’ to the end of the method section and added our 'Patient and public involvement' statement to the end of this paragraph (see also comment (3) of the editor).
3. We have rewritten the first 3 paragraphs.
4. We have decided to skip the summary of the case used, referring to the appendix.

5. We have largely redesigned the sections that relate to the two parts of the research project and the 4 steps that can be distinguished in them. We have not made any changes to the content, only to the design, and believe that it is now much more clearly written and easier for the reader to follow. For that reason, we reformulated the two steps into a primary and secondary goal of the research.
6. The changes to the titles of the 4 steps have also been implemented in the results section.

Changes in the text:

Method

A before-and-after analysis of the quality of the formulated recommendations after sentinel event analysis was conducted during the 2023 SPSC International Patient Safety Conference.
The RIM methodology was trained during a structured 3-hour workshop. For the workshop, the authors used a real sentinel event, as described in appendix 1. Conference participants were invited to attend the workshop, and no selection criteria were applied in the recruitment process. The participants were nurses, medical doctors, pharmacists, dentists, general practitioners, and quality officers. All participants in the workshop were asked about their professional background.

The primary goal was to assess the effect of the RIM methodology in a before and after training setting, with the aim of analyzing its impact on the formulation of higher-quality recommendations, where we hypothesize that the use of the RIM methodology leads to better quality recommendations. The before-and-after training analysis, in which the recommendations formulated by the subgroups before and after training are qualitatively assessed by an expert panel, consisting of 2 experienced trainers in the RIM methodology. The panel uses the RIM methodology to analyze the differences in quality of recommendations before and after the training. The quality of the recommendations is expressed in 1) the filter criteria and 2) the level and category according to the RIM.

The secondary goal was an analysis of the effect of the training itself through an assessment, in which the responses of individual participants are compared with the answers of the expert panel. We hypothesized that the RIM methodology was easy to learn and that the training was usable and feasable.

~~The RIM methodology explained~~

~~The RIM methodology consists of 3 filter criteria and the Recommendation Improvement Matrix (figure 1). The filter consists of three questions, which must be answered positively: 1) is the recommendation well-defined and clear? 2) does the recommendation outline the intended improvement? 3) does the recommendation specify how it will reduce or mitigate the consequences of a similar sentinel event in the future?~~
~~Only recommendations that meet these criteria are subsequently evaluated using the RIM along two axes. The first axis (X-axis) is: does the recommendation prevent the sentinel event from occurring again or does the recommendation focus on mitigating the effects of the sentinel event. The second axis (Y-axis) is the likelihood of the recommendation being able to achieve the effect which is defined. The likelihood that an administrative recommendation is effective is lower than the elimination of the true reason for the occurrence of a sentinel event.~~

~~The research project~~

~~This study does not fall under the scope of the Dutch Medical Research Involving Human Subjects Act (WMO) and therefore does not require approval from an accredited medical ethics committee in the Netherlands. Patients and the public were not involved in the design, conduct, or dissemination of this study. Ethical principles were followed, and at the start of the workshop, all participants were briefed on the study aims,~~

data collection process, and confidentiality measures. Participation in the workshop was considered as implied informed consent, as attendees voluntarily engaged in the study activities. All participant responses were anonymized and stored securely.

The research project consists of two parts:
Part 1. A before-and-after training analysis, in which the recommendations formulated by the subgroups before and after training are qualitatively assessed by an expert panel, consisting of 2 experienced trainers in the RIM methodology. The panel uses the RIM methodology to analyze the differences in quality of recommendations before and after the training.
Part 2. An analysis of the effect of the training itself through an assessment, in which the responses of individual participants are compared with the answers of the expert panel.

All participants in the workshop were asked about their professional background. For the workshop, the authors used a real sentinel event, as described in appendix 1. In summary, the patient had been using coumarin derivatives because of an aortic valve and mitral valve replacement in the past. This medication was discontinued for the placement of a nephrostomy. Two weeks later, the patient developed a stroke, and it was found that the coumarins had not been adequately resumed.
This case was chosen because anticoagulation therapy is used worldwide, and the issues surrounding the prescription of this medication, patient adherence, and especially the temporary discontinuation and resumption of the medication, are globally recognized and recurring problems. The top event, defined as the main incident or adverse event that the analysis seeks to understand and prevent in the future and that represents the point at which control was lost, leading to the incident or near-miss[20], was presented centrally in advance so that everyone could start formulating recommendations from the same starting point. The top event in this case was defined as: 'Anticoagulation treatment has not been adequately resumed'.

Data analysis:
Data were gathered in an Excel database; Excel was used for descriptive statistics. The R statistical software environment version 4.3.2 was used for analysis.

The study was conducted in the following steps (figure 1):

Step 1, Recommendations before training:
Method: After presenting the case, the participants divided into subgroups of a maximum of six people and were asked to draw up recommendations based on the case presented. All recommendations were then assessed through a consensus-based approach by the expert panel. The panel categorized each recommendation as either “did pass or did not pass” filter criteria. For those passing the filter criteria, further categorization was conducted according to the RIM.
Data analysis: For each group, all recommendations were recorded and processed anonymously in a database for scientific purposes. The categorization, as determined by the expert panel, was recorded in both absolute numbers and percentages.

Step 2, Training in RIM methodology and assessment:
Method: After drawing up the recommendations in the subgroups, all participants were trained in the RIM methodology in class using a different case. The learning objective was that the participants were able to primarily align all their concept recommendations with the three filter criteria[19] and then to categorize the concept recommendations that passed the filter criteria according to the RIM methodology[16]. An assessment consisting of eight questions, each with three sub-questions: the filter criteria, the category and level of each recommendation to check whether the material had been sufficiently understood. The test questions were reviewed in advance through a consensus-based approach by the expert panel. The panel identified the most appropriate answers so that the answers of the workshop participants could be mirrored.

Data analysis: The answer to whether a recommendation passes the filter criteria is displayed dichotomously; the answers for the most appropriate level and category according to the RIM are displayed categorically. For all participants, the test results and individual ratings for the training were processed anonymously in a database for scientific purposes. The test results were presented as the mean (SD) percentage of correct answers per participant. To assess whether there was a difference in test scores across professions, a one-way ANOVA was used.

Step 3, Recommendations after training:
Method: After this assessment, the participants were invited to apply what they had learned. In the same subgroups, participants discussed among themselves whether the training in the RIM methodology (i.e. the filter criteria and the RIM) provided grounds to adjust previously formulated recommendations, formulate additional new recommendations if necessary, or remove previously formulated recommendations. In this way, an updated set of recommendations was formulated in each subgroup. All recommendations from all subgroups, both before and after the training, were then consensus-based approached by the expert panel. The panel categorized each recommendation as either "did pass" or "did not pass" the filter criteria and then categorized them according to the RIM.
Data analysis: For each group, all recommendations before and after the training were recorded and processed anonymously in a database for scientific purposes. The quality of the recommendations is expressed in 1) the filter criteria and 2) the level and category according to the RIM. To compare the quality of recommendations before and after the training, the proportion of recommendations that passed the filter were compared with a Chi-squared test.

Step 4, Evaluation:
Method: At the end of the workshop, participants were asked to rate the feasibility and usability of the new method, using the filter criteria and RIM, on a 5-point Likert scale compared to the original method, and to provide explanations and comments about the training.
Data analysis: For all participants, the results of the Likert-scale and individual remarks were processed anonymously in a database for scientific purposes. Results for feasibility and usability were presented as the average value. A t-test was used for comparisons.

The research project
This study does not fall under the scope of the Dutch Medical Research Involving Human Subjects Act (WMO) and therefore does not require approval from an accredited medical ethics committee in the Netherlands. Patients and the public were not involved in the design, conduct, or dissemination of this study. Ethical principles were followed, and at the start of the workshop, all participants were briefed on the study aims, data collection process, and confidentiality measures. Participation in the workshop was considered as implied informed consent**,** as attendees voluntarily engaged in the study activities. All participant responses were anonymized and stored securely. Patients and/or the public were not involved in the design, or conduct, or reporting, or dissemination plans of this research.

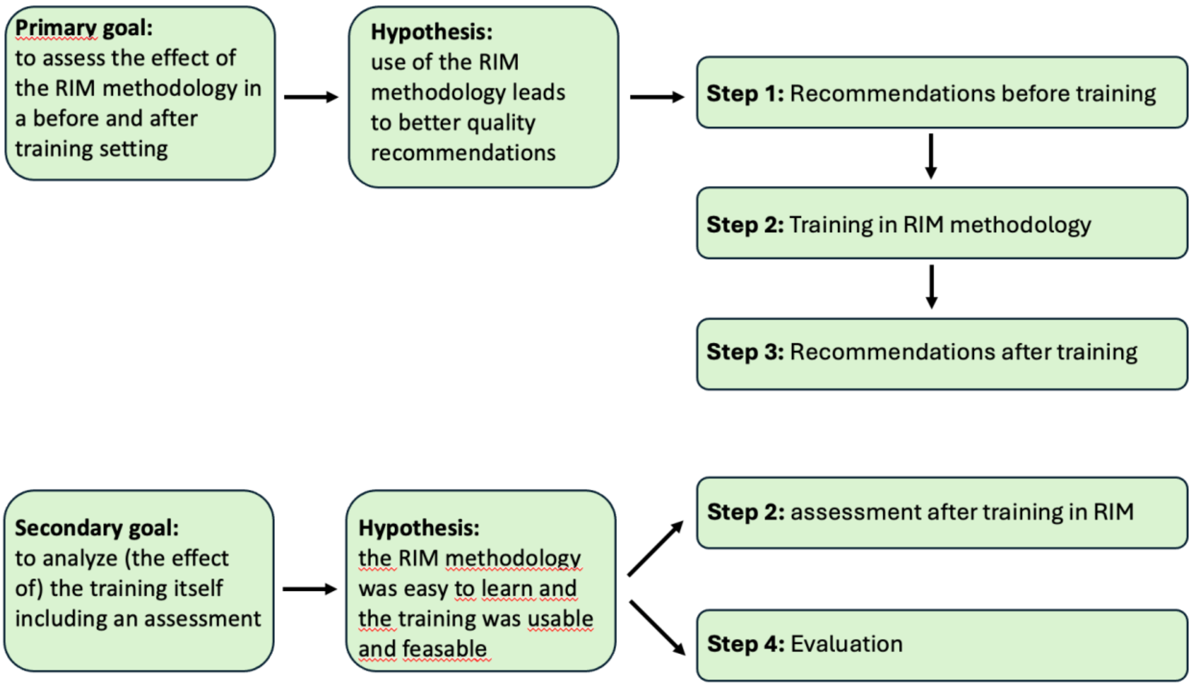

Figure 2 Study design, schematically shown in research goals, associated hypotheses and research steps to be followed.

Comment 3: Discussion
The discussion is short, mainly repeats the results and focuses on describing the strengths and weaknesses of the work. There is no discussion of the results in relation to those published in previous studies. The discussion does not refer to any references. As recommendations for improving safety to prevent a sentinel event are usually made after an in-depth analysis, the question remains to what extent the participants are in a position to make relevant recommendations at all after having read the brief description of the case. This could be discussed as a limitation, too.

Reply 3:
We thank the reviewer for her feedback and agree with her comment that the discussion is short.
Changes in the text:
Discussion
The analysis of the research results shows an improvement in the formulation of recommendations after training in the RIM methodology. Specifically, the results of this study, conducted during a workshop, demonstrated that professionals trained in the RIM methodology: (a) were able to enhance or discard their recommendations after applying the filter criteria and RIM. And (b) were able to assess whether their formulated recommendations would effectively address the sentinel event using the RIM. After training the proportion of recommendations passing the RIM filter increased from 61% to 83% in the paired analysis. These results support our hypothesis that using the RIM methodology leads to better quality recommendations. The results are also in line with our previous findings among inspectors of the Dutch Health and Youth Care Inspectorate and therefore provide additional evidence for the added value of using the RIM methodology[16]. As far as we know, no other studies have been done that have investigated the use of the RIM methodology.

Concerning the secondary goal: an analysis of the effect of the training itself through an assessment, in which the responses of individual participants are compared with the answers of the expert panel

The majority of participants found the RIM methodology both feasible and usable. This is also in line with our previous findings among inspectors of the Dutch Health and Youth Care Inspectorate[16] However, 46% of the participants indicated that additional training was needed. This finding, spontaneously reported by participants of the workshop, was new to us. We do not recognize this from our previous research, but have never actively asked about it. ~~the post-training test results revealed that, on average, participants correctly identified the most appropriate answer for the filter criteria in 61% of the cases in accordance with the expert panel's answers. For the interpretation of the most appropriate Level, this was 16% and for the most appropriate category this was 32%. These findings suggest that a brief training session is sufficient for participants to work with the filter criteria. To further improve the quality of recommendations, additional training should primarily focus on assessing the matrix rather than the filter criteria.~~ As far as we know, no research has ever been done into the effect of such a training itself and these findings lead to new insights.

Limitations
The limited number of participants in this study is undoubtedly a key limitation, which may have contributed to the absence of statistically significant results in some analyses. Moreover, the setting being a conference in which this study was embedded might have influenced the results. Although the time frame for the session was clear and participants were able to attend as the wished. Another possible limitation is the presented case. The case might not have been relevant to all participants. It would be impossible to present a case relevant to all participants. By taking the time and elaborative presenting the case the team tried to mitigate this issue.
Following the training in the RIM methodology, participants changed just over half of their recommendations, indicating that the methodology may have an impact. However, this did not lead to a significant improvement in the level or category of the recommendation as assessed by the RIM. It remains unclear whether this lack of significant upgrades is due to insufficient training on these aspects or because the matrix itself does not inherently facilitate substantial improvement. Further research would be needed to clarify this point. Since recommendations for improving safety to prevent a sentinel event are usually made after an in-depth analysis, it is also debatable to what extent the participants are able to make relevant recommendations at all after reading the brief description of the case and under time pressure.

<u>Comment 4: References</u>
Eight references have been cited in the writing of this article, one of which has been submitted for publication but not yet accepted.

Reply 4:
We thank the reviewer for her valuable comment. We regret to note that this reference (original number 7) was mistakenly included in the list of references. It is a manuscript that was later published under a different title. This reference was already included in the list under number 6. We have removed the reference under number 7 from the list and adjusted the references in the text where relevant. We have also presented the original reference 1 (now noted as reference 2) in more detail.

<u>Comment 5: Minor comment:</u>
The colour scheme used in Figure 2 could be revised. The two intense colours make it difficult to follow the figure. For example, a darker and lighter shade of one colour could be used.

Reply 5:

We thank the reviewer for her valuable comment and in accordance with her suggestion, we have adapted the color scheme of Figure 2 in a darker and lighter shade of the same color.

Changes in the text:

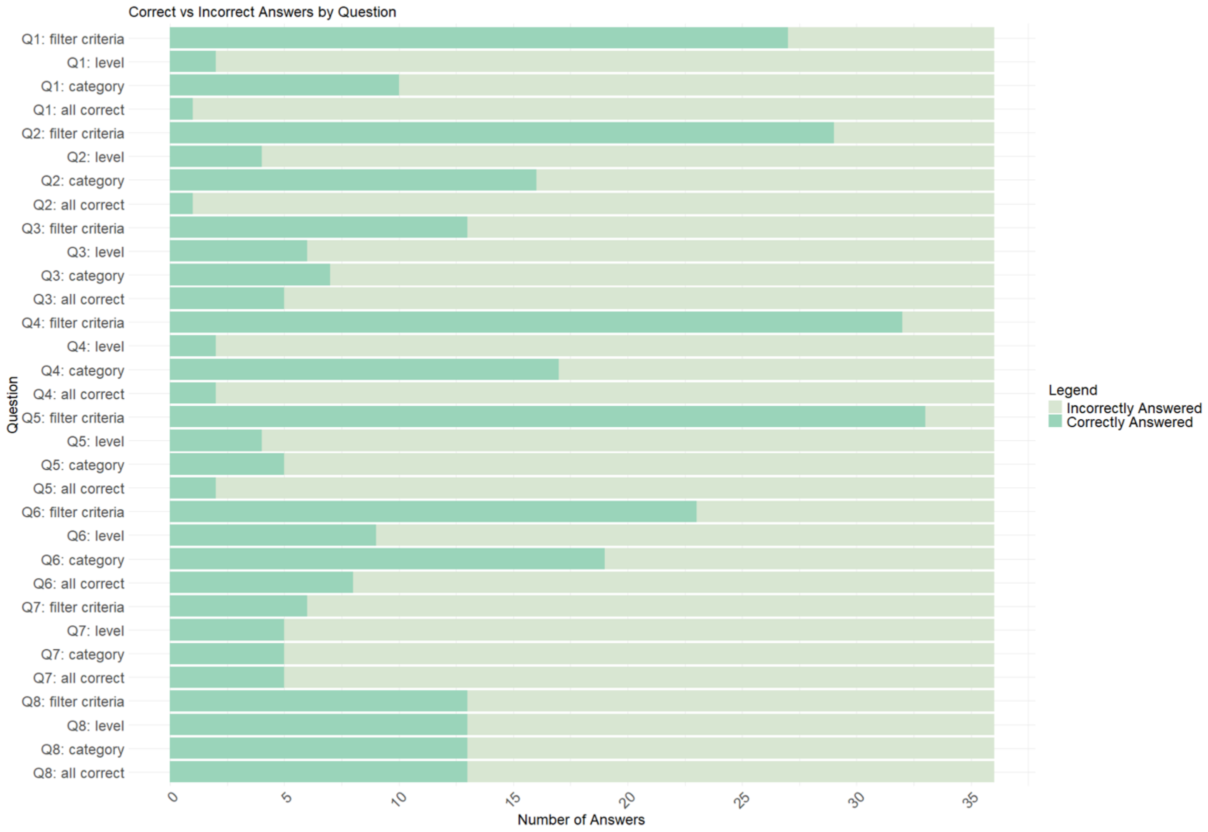

<u>Reviewer 2 Comments</u>

Dr. Luz Berenice López-Hernández , Universidad Autónoma de Guadalajara, Universidad Autonoma de Guadalajara

Comments to the Author:

The authors provide a thought-provoking analysis of a critical gap in sentinel event recommendations—an issue with significant implications for patient safety and healthcare quality. Although the sample size is limited, the study offers meaningful contributions to the field.

<u>Comment 1:</u>

However, the introduction is too brief and lacks sufficient context, which may obscure the study's rationale. Expanding this section to clearly outline the background, significance, and objectives would strengthen the manuscript.

Reply 1:

We sincerely thank the reviewer for her valuable time and effort. We fully agree with her comment and have amended the introduction as follows:

1. To clarify the background, we have added a section in which we describe the most important developments over the past decades that relate to an increasing focus on the development of sentinel event analyses and the effects of the resulting recommendations and added references.
2. We have moved the section explaining the RIM methodology from the method section to the introduction.

3. We have added a paragraph in which we substantiate the rationale behind the study and explain its importance.
4. In the last 2 paragraphs, we have explained the objectives more clearly, as well as the link with the meaning of better future recommendations and thus safer healthcare.

Changes in the text:

Introduction

Sentinel events, or adverse events are defined as unintended patient safety events that result in death, permanent harm or severe temporary harm[1 2]. They are of great concern in health care because the consequences in terms of suffering for patients and their loved ones are immense, the costs are high and same sentinel events keep recurring[3]. Healthcare providers are second victims of such events, which can lead to various effects on the mental healthcare of personnel. For these reasons, it is standard practice to analyze sentinel events and introduce recommendations to prevent new adverse events. In recent decades, there has been an increasing focus on the development of sentinel event analyses and the effects of the resulting recommendations[4-9]. Many studies conclude that recommendations based on sentinel event analyses have only a limited impact on improving quality and safety in health care[4 8 9]. A lack of professionalization of analyses and a lack of standardization may play a role in this, but the quality of recommendations also sometimes leaves much to be desired[4 6 7]. Others point to problems with the implementation of recommendations and make suggestions for this, in which continuity in management and a broader focus on causal factors may be important[5]. Despite this, the same sentinel events continue to occur. In previous research, our group analyzed sentinel events and the recommendations for improvement in all Dutch university hospitals. We concluded that there was a large variety in formulating and selecting of recommendations after sentinel events. Standards for quality assessment and selection of recommendations following the analyses of sentinel events were lacking[10 11].

There are several methods available to prioritize and select recommendations based on assessment of their quality following analysis of adverse events. These methods were primarily developed outside healthcare[12 13]. None of these methods is user-friendly or validated to prioritize recommendations based on objective features[14]. To assess the quality of recommendations from Dutch perioperative sentinel event analyses, we developed a new method that evaluates recommendations meeting the above-mentioned criteria. In previous publications, we have described how we developed the methodology and which methods developed outside of healthcare served as a basis for it[14-17]. Our method was used to assess the quality of recommendations from Dutch perioperative sentinel event analysis reports[15 18]. The so-called Recommendation Improvement Matrix ~~(RIM)~~methodology ~~primarily implies a straightforward approach that uses three filter criteria to determine whether recommendations can be rated as such. Recommendations that meet these criteria are then qualified using the RIM.~~ consists of 3 filter criteria and the Recommendation Improvement Matrix (RIM) (figure 1). The filter consists of three questions, which must be answered positively: 1) is the recommendation well-defined and clear? 2) does the recommendation outline the intended improvement? 3) does the recommendation specify how it will reduce or mitigate the consequences of a similar sentinel event in the future?
Only recommendations that meet these criteria are subsequently evaluated using the RIM along two axes. The first axis (X-axis) is: does the recommendation prevent the sentinel event from occurring again or does the recommendation focus on mitigating the effects of the sentinel event. The second axis (Y-axis) is the likelihood of the recommendation being able to achieve the effect which is defined. The likelihood that an administrative recommendation is effective is lower than the elimination of the true reason for the occurrence of a sentinel event.

We evaluated the applicability of this methodology by analyzing 115 Dutch reports on perioperative sentinel events in which a total of 161 recommendations were made. The RIM proved useful to grade recommendations and was helpful in both designing and selecting recommendations[15]. It was also concluded that the use of the RIM methodology resulted in a fair interobserver agreement (Interclass Correlation Coefficient 0.38; 95% CL 0.20-0.63) and the inspectors involved in the study (n=4) of the Dutch Hthat the RIM is user-friendly, understandable and does not take much time. More importantly they found it potentially useful not only in their own practice but also as a tool for analysing teams to grade their own recommendations[15]. Although the RIM methodology according to this study is promising, the effect of its use on the quality of improvement measures was not yet further studied in a structured way, nor has the impact of structured training in the RIM methodology itself on the ability of healthcare professionals to formulate higher-quality recommendations been extensively evaluated and it was assumed that the tool could be mastered quickly, but whether targeted training, including an individual objective assessment regarding mastery of the method, is necessary to ensure a consistent and correct application has not been investigated before and certainly not in a multidisciplinary context.

~~The authors propose that the next step in preventing the recurrence of similar sentinel events use the adoption of a collaborative and transparent framework to evaluate the recommendations using standardized process grounded in the RIM methodology. Moreover, the effect of training in the RIM methodology has been only briefly described. The authors believe that it is important to properly assess the impact of formal training on the RIM methodology and its influence on the quality of recommendations.~~

The aim of the study was therefore to analyze whether, in a fictitious setting involving a presented sentinel event, participants in a workshop, in which they were trained in the RIM methodology, were able to generate higher quality recommendations using this methodology compared to not using it, as well as to analyze the effects of the training itself, including examination of participants' perceptions of the feasibility and usefulness of the RIM methodology in a healthcare setting.

By addressing these objectives, this study contributes to improving the design of optimal recommendations by integrating the RIM methodology into the routine analysis of sentinel events, which also contributes to safety in healthcare.

Comment 2:

Additionally, the discussion could delve deeper into how the findings compare with existing strategies and should emphasize key takeaways that align with the study's goals. Currently, the document feels unbalanced: the introduction and discussion are too short, while other sections are overly descriptive, disrupting the flow. A more structured and proportionate presentation would improve readability and impact.

Reply 2:

We thank the reviewer for her valuable comment.

We would also like to refer to the answer to reply to comment 3 by reviewer 1. In addition, we discussed how our findings align with both study's goals from our research. We have also expanded the conclusion with suggestions for further research

Changes in the text:

Discussion

The analysis of the research results shows an improvement in the formulation of recommendations after training in the RIM methodology. Specifically, the results of this study, conducted during a workshop, demonstrated that professionals trained in the RIM methodology: (a) were able to enhance or discard their recommendations after applying the filter criteria and RIM. And (b) were able to assess whether their formulated recommendations would effectively address the sentinel event using the RIM. After training the proportion of recommendations passing the RIM filter increased from 61% to 83% in the paired analysis. These results support our hypothesis that using the RIM methodology leads to better quality recommendations. The results are also in line with our previous findings among inspectors of the Dutch Health and Youth Care Inspectorate and therefore provide additional evidence for the added value of using the RIM methodology[16]. As far as we know, no other studies have been done that have investigated the use of the RIM methodology.

Concerning the secondary goal: an analysis of the effect of the training itself through an assessment, in which the responses of individual participants are compared with the answers of the expert panel
The majority of participants found the RIM methodology both feasible and usable. This is also in line with our previous findings among inspectors of the Dutch Health and Youth Care Inspectorate[16] However, 46% of the participants indicated that additional training was needed. This finding, spontaneously reported by participants of the workshop, was new to us. We do not recognize this from our previous research, but have never actively asked about it. ~~the post-training test results revealed that, on average, participants correctly identified the most appropriate answer for the filter criteria in 61% of the cases in accordance with the expert panel's answers. For the interpretation of the most appropriate Level, this was 16% and for the most appropriate category this was 32%. These findings suggest that a brief training session is sufficient for participants to work with the filter criteria. To further improve the quality of recommendations, additional training should primarily focus on assessing the matrix rather than the filter criteria.~~ As far as we know, no research has ever been done into the effect of such a training itself and these findings lead to new insights.

Strengths & limitations

Strengths

This is the first study in which the same case and the same top event have been used as a starting point by all study multidisciplinary participants to investigate the effects of the RIM methodology on the quality of recommendations. This contrasts with our previous studies, in which each subgroup of participants worked with recommendations from cases they had recently analyzed themselves. These recommendations were then reassessed by the participants after training in the RIM methodology to evaluate the effect on the quality of those recommendations. In those studies, each subgroup worked based on its own set of recommendations.

Due to the design of this study, which ensured the same baseline values for each participant, a more robust before-and-after analysis could be conducted. The results indicate that after the training, participants demonstrated a strong ability to correctly identify valid recommendations, as evidenced by a sensitivity of 86%. This suggests that the training effectively enhanced their ability to recognize recommendations meeting the established filter criteria. However, the specificity of 71% indicates that there is room for improvement in accurately identifying recommendations that do not pass the filter. The fact that the participants were multidisciplinary adds to the generalizability of the results.

In previous research, we employed the RIM methodology, but did not adequately assess whether participants had truly mastered its use. Our current results indicate application of the filter criteria requires only limited training to achieve an improvement in the quality of recommendations. However, proper use of the RIM requires more extensive training. Nonetheless, it appears feasible to further enhance the quality of recommendations with additional training.

At the end of the workshop, all participants were asked to rate, using a 5-point Likert scale, the feasibility and usability of the RIM methodology, compared to the originally used method. Participants were also invited to explain their scores and provide comments on the training received. Of the 24 participants, 11 (46%) spontaneously indicated a need for additional training. Notably, no comments were made that discredited the quality of the training. This suggests that the training itself was adequate; however, there was insufficient time for participants to fully comprehend the material. Providing more time to practice, particularly with the matrix, appears to offer significant added value.

The need for additional training is further supported by the post-training test results, which revealed that only 13% of participants provided the most appropriate answers as determined by the expert panel. In our previous studies, we did not explicitly test whether the training was adequately understood. This aspect warrants attention in future research. It is plausible that enhanced training, coupled with the implementation of a robust assessment framework to evaluate comprehension of the material, could further amplify the benefits of the RIM methodology.

Also noteworthy in this context is the relationship between the expressed need for additional training and the post-training test results among the group of nurses. Although the group consisted of only six responding participants, only two nurses (33%) indicated a need for more training, despite this group achieving the most appropriate answers in the post-training test in only 9% of cases. This discrepancy suggests a potential gap between the perceived need for further training and the actual requirement based on performance. These findings support the importance of implementing a robust assessment framework to objectively evaluate comprehension of the material.

The evident need for additional training warrants careful consideration in future research and should be accounted for in the interpretation of the findings of this study. Enhanced and more comprehensive training has the potential to further improve the formulation of optimal recommendations. A recommended approach to achieving this could involve incorporating practice with multiple cases, accompanied by focused discussions. Training should prioritize the assessment and application of the RIM, while placing comparatively less emphasis on the filter criteria, which appear to require less extensive instruction.

Limitations

The limited number of participants in this study is undoubtedly a key limitation, which may have contributed to the absence of statistically significant results in some analyses. Moreover, the setting being a conference in which this study was embedded might have influenced the results. Although the time frame for the session was clear and participants were able to attend as the wished. Another possible limitation is the presented case. The case might not have been relevant to all participants. It would be impossible to present a case relevant to all participants. By taking the time and elaborative presenting the case the team tried to mitigate this issue.

Following the training in the RIM methodology, participants changed just over half of their recommendations, indicating that the methodology may have an impact. However, this did not lead to a significant improvement in the level or category of the recommendation as assessed by the RIM. It remains unclear whether this lack of significant upgrades is due to insufficient training on these aspects or because the matrix itself does not inherently facilitate substantial improvement. Further research would be needed to clarify this point. Since recommendations for improving safety to prevent a sentinel event are usually made after an in-depth analysis, it is also debatable to what extent the participants are able to make relevant recommendations at all after reading the brief description of the case and under time pressure.

The observed request and perceived need for additional training was a new phenomenon in this study. In previous studies, we did the general supposition that the RIM methodology could be learned easily and in a short time.
A potential explanation for the discrepancy between earlier experiences and the current findings could be the diverse professional backgrounds of the participants. In addition to medical doctors and quality officers, the participant pool included nurses, pharmacists, and dentists. In our prior research, participants were exclusively medical doctors and/or quality officers.
The case study employed in this workshop was based on a hospital setting, which may have influenced the results. Dentists, in particular, may have been less familiar with the pathology described in the case, potentially affecting their engagement and performance during the training. Another explanation for this discrepancy between previous experiences and the current outcome could be that the training and the test were offered in English, while for the participants as well as the trainers this was not their native language.

Recommendations
We recommend extending the training to more than one day, piloting the revised format, and comparing it to the current structure to measure usability and impact. This additional time would enable all participants to develop a shared understanding of the material. A longer duration would allow for comprehensive coverage of key topics, interactive discussions, and hands-on practice, ensuring that participants with varying levels of prior knowledge can effectively grasp the concepts.
Such an extended format would foster a more inclusive learning environment and enhance the overall impact of the workshop. Furthermore, we suggest incorporating this workshop into the medical curriculum or offering it as a highly recommended Continuing Professional Development (CPD) program. This approach would help ensure that healthcare professionals are equipped with the necessary skills and knowledge to keep pace with advancements in the field.

Conclusion

Analysis of the research results show an improvement in the formulation of recommendations following training in the RIM methodology.
Specifically, the findings from this workshop indicate that professionals trained in the RIM methodology were able to:
a) Evaluate whether their formulated recommendations were structured in a way that would effectively address the sentinel event using the RIM methodology's filter criteria, and
b) Enhance or discard recommendations as needed based on the RIM methodology.
The majority of participants found the RIM methodology both feasible and usable. However, a significant proportion also expressed the need for additional training. While the training successfully improved participants' ability to identify valid recommendations, further efforts are necessary to enhance their capability to accurately identify and address non-compliant recommendations. This would help minimize the inclusion of potentially weak recommendations in future assessments.
It is plausible that with more comprehensive training, particularly with a stronger emphasis on the RIM, the ability to formulate optimal recommendations could be further improved, amplifying the overall effectiveness of the RIM methodology. Future research could be a cluster-randomised or stepped-wedge study comparing RIM training with usual practice. This research should examine learning curves, retention, and training dose–response. Psychometric evaluation of the Matrix is needed to improve reliability and validity. And most important linking RIM quality to implementation success and patient safety outcomes is essential.

VERSION 2 - REVIEW

**Reviewer**	**1**
**Name**	**Polluste, Kaja**
**Affiliation**	**University of Tartu, Department of internal Medicine**
**Date**	**10-Oct-2025**
**COI**	

Thank you for the opportunity to read the revised manuscript. The authors have carefully followed the editor's and reviewers' suggestions and significantly improved their manuscript. I have no further suggestions.
